# Development and Validation of the Purity Orientation–Pollution Avoidance Scale: A Study With Japanese Sample

**DOI:** 10.3389/fpsyg.2021.590595

**Published:** 2021-02-11

**Authors:** Hideya Kitamura, Akiko Matsuo

**Affiliations:** ^1^Department of Sociology, Toyo University, Bunkyo, Japan; ^2^Department of Psychology, Tokai Gakuen University, Nagoya, Japan

**Keywords:** morality, moral foundation theory, Japan, purity, culture

## Abstract

The moral foundations theory (MFT) proposes that there are five moral foundations that work as the standard to make moral judgments. Among them, the purity foundation is a complex concept. It is considered to be a distinctive foundation compared with the other ones partly because it involves religious beliefs. The assumption underlying the purity foundation is Christian beliefs, so the MFT was developed and made prevalent mostly in the Western cultures. However, because of that assumption, cultural differences in perceiving the purity foundation should be observed with a non-Western sample, such as Japan. It would be important to discuss and clarify the Japanese unique aspect of their orientation toward the pure and impure. We constituted a scale to measure people's tendency toward purity orientation–pollution avoidance (POPA), based on the purity/sanctity subscale of the MFT. For validation, we administered several scales along with POPA. In Study 1, we developed the scale and measured the relationship between the degree of one's POPA, disgust, and animism. We identified four factors as POPA subscales. In Study 2, we investigated the test–retest reliability of POPA and conducted questionnaire surveys to measure attitudes toward paranormal phenomena and the degree of concern for each of the moral foundations. The results showed the validity of the scale, based on the moderate correlations with other scales. The POPA can be a promising tool to better understand the phenomena involving the purity foundation in a Japanese context.

## Introduction

Research on morality has been increasing significantly, with such studies gaining significance in a conflict-ridden world, given the growing disputes between and within countries and even districts. In recent times, the literature on the standard of moral judgment has been accumulated since the intuitionist model of moral judgment argues that people's feelings of wrongness come first and the rationale for the judgment follows. According to certain proposed theories about moral standards, people have a fixed number of modes of reasoning about moral judgment, which is called moral pluralism. These multiple ethics guide moral judgment by providing a template for understanding and interpreting potentially moral-relevant behavior. As a result, people reach conclusions about the rightness or wrongness of a specific behavior by evaluating it with respect to one of the ethics.

Shweder et al. (1997) highlighted three important components to provide a reason for a moral-relevant situation by investigating the moral standards in the Western and Eastern cultures. Based on the idea of moral pluralism, they advocated autonomy, community, and divine ethics in moral judgment. The authors argued that community and divinity have an especially significant status in the Eastern cultures. Divinity provides an important value for religious purity. Graham et al. (2013) advocated a theory called moral foundations theory (MFT), which states that people utilize the domains of care, fairness, group, authority, and purity to make moral judgments. As a theory that was extended from Shweder's three ethics, MFT identifies care and fairness moral foundations as the components of the autonomy ethic pertaining to individual rights and justice. Ingroup and authority moral foundations are the components of the community ethics, which are sensitive to one's membership of groups with hierarchy. Divinity ethic is essentially equivalent to the purity foundation. By applying one of these three ethics to potentially moral violating situations, people recognize whether those situations are right or wrong and understand why they are so.

While Haidt (2012) considered moral foundations as Shweder's three major components by connecting care and fairness, and loyalty and authority, the authors perceive purity as an independent factor in moral judgment. Kaur and Sasahara (2016) effectively analyzed tweets using latent semantic analysis and found that purity is a unique component independent of the other foundations. The purity foundation is reported to be relevant to disgust, which serves to avoid danger from food poisoning and subsequently preventing illness (Rozin et al., 1999; Van Leeuwen et al., 2012). Therefore, it is reasonable to state that moral purity is also relevant to purity of the body or cleanliness (Zhong and Liljenquist, 2006). Beyond physical cleanliness, purity is related to religiosity (Graham and Haidt, 2010; Preston and Ritter, 2012). In addition, purity involves sexuality or sexual behavior. In the olden days, people considered sexual behavior as offensive and were not willing to discuss topics concerning sex, especially in public, because it was considered a taboo. Douglas (2003), an anthropologist, reviewed the idea of impurity (i.e., the opposite idea of purity) and argued that the impure consisted of the representations of death, blood, sexuality, and body.

Based on Douglas's claim (2003), purity can further involve respect for the substantially invisible, such as sanctity, that does not necessarily rely on a clear ground for their existence. Respect for the invisible has been eagerly investigated in the recent literature on awe. Awe is defined as an emotional response to vast perceptual stimuli that update one's cognitive framework (Keltner and Haidt, 2003). Awe is often induced by pictures or videos depicting magnificent landscapes (e.g., mountains and universe) (Shiota et al., 2007). Through the experience of being in awe of nature, the psychological state of purity could be accomplished among the Japanese people (Ishikawa, 2012). This psychological purity is regarded as a feeling of cleanness and rejuvenation, which is theoretically in accordance with the value of purity.

Purity has not yet been well-defined because cultural differences can exist in the context of purity. Specifically, the representations of purity can vary depending on culture, which is the reason why researchers find it difficult to measure purity. Christianity, which is a popular religion in the West, is not so prevalent in the Asian cultures. Asian countries, such as Japan, often follow the culture of Buddhism or polytheism, or a mixture of both. As mentioned above, purity as a virtue has a central idea of sacredness, but sanctity represented in theoretical purity may be limited to Christianity. Conversely, the violation of purity or impurity is considered to be associated with infection and sexual components (Koleva et al., 2012; Van Leeuwen et al., 2012). However, these concepts have not been tested in relation to the purity foundation in the Asian cultures. As a result, the purity items on the questionnaires that measure one's importance of moral foundations may not be directly applied to Asian participants (Iurino and Saucier, 2018). The investigation of the culturally specific purity foundation would be beneficial. Because variations in results from research on social psychology theories have been observed even inside the “Asian groups” (e.g., East Asians vs. South Asians; Gelfand and Denison, 2020), cultural differences in the perception of purity would be present across the Asian cultures. Thus, we specifically look into the purity foundation in Japan in the current research because the sense of purity is not systematically organized or investigated in the field of psychology in the Japanese culture.

Impurity (*kegare* in Japanese) and people's tendency to avoid impurity, rather than purity itself, has been historically discussed in diverse fields in Japan (e.g., religion and anthropology) and interwoven in various contexts (e.g., cultural and historical) (e.g., Miyamoto, 2008; Namihira, 2009). In Japan, impurity is a phenomenon that includes double aspects, one of which comes from Shintoism and Buddhism, and the other is a theoretical framework used in academic fields. The former aspect is similar to Douglas's claim about (im)purity, arguing that the nature of impurity is disorder and that impurity is a matter about something out of place (Miyamoto, 2008). While Douglas (2003) based her theoretical construction mainly on Judaism, Japanese impurity in this sense derives from Shintoism and Buddhism (often in a mixed form). Natural disasters, death, and blood (and anything that symbolizes them) are viewed as impurity. Impurity is infectious and can be removed through appropriate rituals and passage of time (Ito, 2002).

As mentioned above, religion is an essential element of purity. The Japanese people do not affiliate themselves with a religion, but they are not non-religious. The characteristics of religion for the Japanese are different from those of the West to a large degree. Although the Japanese people do not have specific religious dogmas or groups, they do have something to believe in from ancient times. That target can be understood as nature worship, but it is a naive feeling since ancient times and concerns animism. Of course, there is no such religion as an animism sect; thereby, the naive feeling is not organized as a religious community or clarified through scriptures. Thus, believers themselves are not likely to be sure of their own dogmas and disciplines inside them.

However, some traditional institutions, such as shrines symbolized by Shintoism and temples symbolized by Buddhism, are somewhat rooted in the Japanese people's minds and embody their religious spirits deep inside. Because most of the Japanese people do not consciously believe in a specific religion with its clear dogmas, they cannot even discriminate Shintoism from Buddhism. As a result, they mix up Shintoism and Buddhism, but the fact of their mixing itself is part of the Japanese tradition since 1,000 years ago (Gokure, 2009).

Douglas's theory is criticized for her full application of purity as the Jewish symbolization to people's real life (Miyamoto, 2008), but Japanese researchers (albeit outside psychology) have attempted to sophisticate the religious meaning of impurity to use as a framework explaining practical situations in everyday life in Japan (the latter aspect of Japanese purity described above). In addition to the religion-oriented sense of impurity, social sense of impurity in Japan includes something out of order and the process surrounding it—moving from one place to another over its boundary (e.g., saliva in one's mouth is not impure because it is inside its appropriate place. However, once s/he lets that saliva out of the mouth, the saliva itself and the process to let it out become impure) (Usui, 1986; Baba, 2000). One important point about impurity in Japan is that even “pure” targets can be degraded when they are moved from their original place (i.e., the behavior to take a pebble from the territory of a shrine and the pebble itself after the behavior are both impure). Thus, avoidance of such impurity has social meaning in these days (Tanaka, 2004). Further, because impurity is believed to be removed through rituals to obtain the status of purity, purity is often associated with something “good” and impurity is with “bad” (Baba, 2000; Xu, 2013). That can be why (im)purity became a part of morality, at least in Japan. However, those arguments have been not of interest in psychology.

In sum, previous research conducted in the West has found that the concept of purity is symbolically associated with religiosity, physical cleanliness, sexual behavior, and illness. We developed a purity scale that can be applied to the Japanese people by referring to the Moral Foundations Questionnaire (MFQ) as an existing framework, which measures moral foundation from the Western perspective. The theoretically opposite concept of purity can be defined as pollution. Pollution is an attitude that is wary of and avoids contamination, dirt, and infection. The scale we developed features the core items involving animism and respect for shrines/ temples, along with the other items about taboos of body and sexual matters and threats of diseases and impurity caused by infection. To test the validity of our purity scale for the Japanese people, we measured one's level of animism (one's belief that natural objects possess minds and spirits and one's worship of nature involving awe), one's familiarity with and the tendency to believe in non-scientific supernatural phenomena, and the emotion of disgust that has been found to be associated with pollution. We report here how we developed our purity scale among the Japanese [the purity orientation–pollution avoidance (POPA)] and tested its validity and reliability.

## Study 1

The POPA was developed, and we identified the items, confirmed the factors, and validated it using the Japanese version of the Disgust Scale-Revised (DS-R-J) (Iwasa et al., 2018) and Animism Scales (Ikeuchi, 2010). The POPA should be relevant to disgust and Haidt's to moral foundations. Disgust is found to be relevant to religiosity, personal hygiene, and illness. Since we needed to find the difference between the concepts of disgust and avoidance of pollution (i.e., impurity), the DS-R-J and Animism Scales were included to investigate the validity of our POPA. It was predicted that participants with a high score of purity orientation would have high scores of disgust sensitivity and animism orientation. However, because one's purity orientation involves the feeling of psychological pollution that is different from physical pollution, the correlation between the POPA and disgust sensitivity would be not very high but moderate. Likewise, animism involves the idea that one views an inanimate object as the other self of himself/herself, which is different from one's purity orientation. Thus, the correlation between the POPA and animism would be moderate, as well.

## Methods

### Participants

Participants included 411 Japanese undergraduates enrolled in introductory psychology classes at a major Japanese private university, of whom 229 were males. They agreed to participate in the study for extra credit or credit toward a course research requirement. There was no financial incentive provided to the participants. The survey was delivered online with Google Forms. Ethical review and approval by the ethics committee of Toyo University (P18033) were obtained before conducting this study. The mean age of the participants was 19.50 (*SD* = 1.40).

### Measures

#### Purity Orientation–Pollution Avoidance

A sample of undergraduate students from Japan (*N* = 63) freely generated situations in line with each of Shweder's three ethics, as part of a different cross-cultural project. In this process of situation collection, the definitions of the three ethics were first explained to the participants, and they were asked to describe as many moral violation situations as possible that they had experienced, heard, or imagined for each domain. All sessions were conducted in a small-group setting that consists of one to three participants in the same room for 20 min (cf. Morling et al., 2015).

These definitions were developed by the second author, based on the works of Shweder and Haidt (Shweder et al., 1997; Haidt, 2012). After creating the first draft, she discussed whether the descriptions are appropriate in accuracy and length with two researchers specializing in morality research. Because the descriptions were needed to be reasonably easy to understand and imagine to lay people, the second author then revised the original drafts based on feedback received from two undergraduate students. The final version of the descriptions presented to the participants was as follows:

Ethic of Autonomy: This perspective is based on the idea that people are, first and foremost, autonomous individuals with wants, needs, and preferences. People should not prevent each other from satisfying these wants, needs, and preferences as they see fit. When people do not interfere too much in each other's projects and respect each other's opinions and choices, societies develop moral concepts such as rights, liberty, and justice.

Ethic of Community: This perspective is based on the idea that people are, first and foremost, members of communities such as families, teams, armies, companies, tribes, and nations. People should not disturb the social order. When each individual fulfills the role/duty assigned to him/her, societies develop moral concepts such as duty, hierarchy, tradition, respect, and reputation.

Ethic of Divinity: This perspective is based on the idea that people, first and foremost, exist with the spiritual realm and are connected to the divine soul beyond our secular world. People should therefore not degrade or dishonor their own spirit, that of others, or the sanctity of the natural order of things. Even when one is fulfilling his/her role /duty to his/her community, she/he is not allowed to behave in a disgusting way. When people protect themselves and others from degradation, societies develop moral concepts such as sanctity, elevation, and purity.

Based on the 257 descriptions of divinity violations collected, we prepared the items mainly relevant to (1) religiosity, (2) death, (3) nature worship, (4) body, (5) sexual issues, and (6) disgust of cleanliness and uncleanness. Those items include such Japanese unique customs as seasonal grave visiting. Some examples included “to scribble on the shrine wall” and “to insult the dead.” We considered that the purity foundation among the Japanese includes psychological purity as an important factor in addition to physical purity (i.e., cleanliness) because the Japanese would think that it must be a way of attaining purity in their mental state. For example, many Japanese believe that standing under a waterfall at the river in the mountain is a powerful and effective way of attaining purity (cf. Uehara, 2017, for the Japanese people's cleanliness). Overall, 48 items were prepared to measure purity orientation and the likelihood to avoid pollution. Each item was rated on a Likert-type scale, ranging from 1 (not at all/almost never) to 7 (very much/almost always) (1 = 全くそう思わない, 2 = ほとんどそう思わない, 3 = どちらかと言えばそう思わない, 4 = どちらとも言えない, 5 = どちらかと言えばそう思う, 6 = わりあいそう思う, and 7 = かなりそう思う in Japanese). The materials, including the whole questionnaire, are available online at https://osf.io/keuwd/.

#### Disgust

The DS-R-J (Iwasa et al., 2018) Scale measures the degree of an individual's reaction to disgust elicitors with 18 items. The disgust elicitors are categorized into three subscales: core disgust (CD; eight items), animal reminder disgust (AR; five items), and contamination disgust (CO; five items). The DS-R-J Scale was found to have a sufficient level of reliability and validity with a Japanese sample. Some sample items include “It would not upset me at all to watch a person with a glass eye take it out of the socket” and “You are about to drink a glass of milk when you smell that it is spoiled.”

#### Animism

The Animism Scale (Ikeuchi, 2010) is a self-report measure to quantify one's animism, which is defined as “the phenomenon that people perceive the existence of life and/or God in inanimate objects, even though they know that these objects are not actually alive.” This scale has 14 items, and it was found to comprise three factors. Some sample items include “I sometimes give names to the objects around me” and “I believe in multiple gods, such as the god of mountains and another god of the ocean.”

### Procedure

The participants were informed of the link to the above questionnaires during the class where they were offered the opportunity to earn extra credit points for their participation. They anonymously responded to the three scales, POPA, DS-R-J, and Animism, online outside the class. We administered another scale, but it was not relevant to this study. The order of the questionnaires was counterbalanced.

## Results

Examining the scree plot, we performed an exploratory factor analysis for POPA and obtained loadings using the principal factor method with oblimin rotation. Twenty-one items were excluded because of low factor loadings for all four factors (<0.40), and each of their loadings did not belong to one factor. The Kaiser–Meyer–Olkin (KMO) measure and Bartlett's test were conducted to determine whether they could be further analyzed. The KMO measure of sampling adequacy figure was 0.88, which shows the adequacy of the sample. Bartlett's test of sphericity value was 5,462.83, *p* <0.001. This shows that it was feasible for further processing. We then conducted a revised exploratory factor analysis. As a result, the original 48 items were reduced to 27 items. Four factors were found, as shown in Table 1.

**Table 1:** Revised exploratory factor analysis of the purity orientation–pollution avoidance (POPA) items (*N* = 411).

**Item**	**Wording**	**F1**	**F2**	**F3**	**F4**	***h^**2**^***
6	I think that mountains, rivers, and rocks posses divinity that is connected to God.	**0.68**	0.19	−0.32	−0.06	0.46
27	I think that my body would be clean after I soak myself into clean water.	**0.63**	−0.12	0.10	−0.06	0.40
26	I think that exposing myself in a waterfall at the river in the mountain is a powerful and effective way of acquiring purity mentally.	**0.58**	−0.07	0.11	−0.06	0.34
28	I think that keeping my body clean is effective for calming down.	**0.53**	−0.01	0.11	0.01	0.28
34	I think that my family experience something terrible if I never visit my ancestors' graves.	**0.52**	0.20	0.14	−0.05	0.27
7	I think that nature will show its fury if human beings continue environmental destruction.	**0.48**	0.22	−0.05	0.07	0.23
5	I strongly feel in awe of the greatness of nature.	**0.47**	0.01	−0.16	0.09	0.22
46	I think that my mouth gets corrupted by telling a lie.	**0.46**	−0.01	0.19	0.03	0.21
47	I feel like getting a fresh start and revitalized mentally and physically when a new year has come.	**0.44**	0.14	0.04	0.01	0.19
32	I think that I will be cursed if I litter inside the grounds of a temple and shrine.	0.15	**0.77**	−0.03	0.01	0.59
31	I think that I will be cursed if I steal the offerings placed at graves.	0.1	**0.73**	−0.03	−0.02	0.53
19	I feel uncomfortable putting amulets and talismans from last year in the trash cans.	−0.04	**0.62**	−0.07	0.02	0.38
35	I never want to live in an apartment where someone killed him/herself.	−0.13	**0.55**	0.13	0.12	0.30
1	I think that it is inexpiable to make religious institutions (such as a shrine) dirty.	0.19	**0.47**	0.04	−0.01	0.22
20	I feel cordiality from the amulets given by someone.	0.14	**0.43**	−0.04	−0.06	0.18
22	I think that inappropriate sexual behavior makes my soul dirty and leads to an unhappy life.	0.05	−0.09	**0.73**	0.01	0.53
23	I think that vicious thoughts make my soul dirty and leads to an unhappy life.	0.24	−0.13	**0.67**	0.02	0.45
21	I think that sex drive makes my soul dirty.	−0.05	−0.11	**0.6**	−0.01	0.36
30	I think that cheating and adultery make my body and soul dirty.	−0.21	0.38	**0.53**	−0.04	0.28
43	It is dirty to have sexual relationship with a biologically close individual.	−0.12	0.19	**0.47**	0.04	0.22
29	I think that going on a compensated date makes my body and soul dirty.	−0.18	0.33	**0.47**	−0.03	0.22
37	It is inexpiable to walk on the street naked.	−0.03	0.24	**0.43**	−0.15	0.18
13	I feel uncomfortable sharing a large platter or a hotpot with other people.	0.01	−0.18	−0.04	**0.69**	0.48
2	I don't want to use the mug that someone else uses regularly, even after it is sanitized.	−0.03	−0.05	0.10	**0.56**	0.31
12	Although some people can eat the food that is dropped on the ground, I can never do it.	−0.06	0.14	−0.06	**0.55**	0.30
10	I want to go elsewhere if someone next to me on the train seems to have a cold and coughs terribly.	−0.19	0.25	0.01	**0.46**	0.21
11	I feel uncomfortable if I do not wash my hands before meals.	0.14	0.15	−0.05	**0.44**	0.19
	Correlation matrix among rotated factors					
		1.00	0.56	0.49	0.29	
			1.00	0.46	0.11	
				1.00	0.26	
					1.00	

*Bold values represent factors. F1, Mental Purity; F2, Respect for Religion; F3, Bodily Purity; F4, Pathogen Avoidance*.

The first factor indicating mental purity was named “Mental Purity” and was represented by nine items. The second, named “Respect for Religion,” was relevant to religious and deviant behavior at religious facilities. Six items were strongly associated with the second factor. The third factor, represented by seven items, involved bodily matters and sexual behavior, and it was named “Bodily Purity.” Finally, the fourth factor, represented by five items, was found to be relevant to pollution and the lack of cleanliness as well as concerning illness. We named this fourth factor “Pathogen Avoidance.” Internal consistency was sufficient for all factors (Cronbach's alpha = 0.814, 0.808, 0.781, 0.661).

Through a series of exploratory factor analyses, we found three factors for the Animism Scale, which were suggested as the original factor structures. We performed an exploratory factor analysis for the DS-R-J Scale. The analysis resulted in three factors, but the composition of the items was different from the results derived by Iwasa et al. (2018). According to the factor structure obtained in our factor analysis, we employed a different set of three subscales from the original structure. Those three subscales were named “Dead body,” “Corruption,” and “Avoidance of uncleanness” (available online at https://osf.io/keuwd/). We calculated the means of the responses to the items belonging to each subscale for the DS-R-J and Animism Scales. The correlations between the subscales are presented in Table 2.

**Table 2:** Purity orientation–pollution avoidance (POPA) correlations with convergent and discriminant measures (*N* = 411).

**Scale**	**Subscale** ** (*M*, *SD*)**	**Respect for Religion**	**Bodily** ** Purity**	**Pathogen Avoidance**	**Dead** ** body**	**Corruption**	**Avoidance of uncleanness**	**Apotheosis of natural products**	**Parts of possessors**	**Anthropomorphication of possessions**
POPA	Mental purity (3.87, 1.17)	0.537^**^	0.469^**^	0.210^**^	0.255^**^	0.221^**^	0.352^**^	0.622^**^	0.439^**^	0.440^**^
	Respect for religion (5.47, 1.19)		0.458^**^	0.158^**^	0.458^**^	0.370^**^	0.413^**^	0.463^**^	0.523^**^	0.361^**^
	Bodily purity (4.02, 1.11)			0.194^**^	0.361^**^	0.328^**^	0.438^**^	0.256^**^	0.217^**^	0.162^**^
	Pathogen avoidance (3.64, 1.23)				0.217^**^	0.201^**^	0.387^**^	0.091^*^	0.052	0.064
DS-R-J	Dead body (3.87, 0.86)					0.463^**^	0.519^**^	0.092^*^	0.114^*^	0.020
	Corruption (4.45, 0.63)						0.483^**^	0.127^**^	0.189^**^	0.142^**^
	Avoidance of uncleanness (3.21, 0.83)							0.189^**^	0.182^**^	0.128^**^
Animism	Apotheosis of natural products (2.85, 1.15)								0.498^**^	0.489^**^
	Parts of possessors (3.76, 0.88)									0.643^**^
	Anthropomorphi-cation of possessions (3.12, 0.86)									

* *p < 0.05*;

** *p < 0.01*.

As expected, the subscales of POPA are weakly and moderately correlated to all the subscales of the Disgust Scale (ranging from *r* = 0.201–0.458). They are also weakly and moderately correlated to the subscales of the Animism Scale after the items that were similar to those in POPA (ranging from *r* = 0.052–0.622) were eliminated. The “Pathogen Avoidance” in the POPA was weakly correlated to both the Disgust and Animism Scales, which makes this factor different from the others.

## Discussion

We developed and validated the POPA. Japanese purity orientation, which plays an important role in their value system, should be related the most to and reflected in the “Mental Purity” subscale of the POPA because the “Mental Purity” subscale represents one's tendency to actively pursue values in something pure. Because purity orientation among Japanese implies worship, that orientation should theoretically involve the “Respect for Religion” subscale, as well. The “Respect for Religion” subscale was found to include both items of a positive aspect and a negative aspect: the attitudes toward being engaged in religious rituals to mourn the deceased and the sense of avoiding the pollution of the sacred. Accordingly, the “Respect for Religion” subscale seems to involve both Japanese purity orientation and pollution avoidance, which is not just about religious worship. Because the ideas underlying the “Bodily Purity” and “Pathogen Avoidance” are about being polluted in a physical sense, those subscales should be related to the pollution avoidance aspect of Japanese purity orientation. The “Pathogen Avoidance” subscale involves one's health in a practical sense and represents a sense of avoiding dirty and unhygienic conditions, unlike the “Mental Purity” subscale. The “Pathogen Avoidance” subscale represents passively avoiding pollution in a body-related term, so that this subscale features a slight difference from the other subscales that all involve religious and psychological factors to a greater or lesser degree. The “Bodily Purity” subscale of the POPA tended to show a relatively high correlation to each of the subscales of the DS-R-J Scale. Pollution avoidance is basically an attitude against something dirty; its relationship with the reaction to disgust elicitors (which is measured by the DS-R-J Scale) demonstrates the validity of POPA. Further, all the POPA subscales, including “Bodily Purity,” are not highly correlated with the DS-R-J Scale because pollution avoidance is the feeling to seek psychological cleanliness, which is more than just physical hygiene. This correlation indicates that pollution avoidance is a different feeling from disgust and an independent psychological phenomenon of disgust, which demonstrates the validity of pollution avoidance as a concept and of the scale to measure the concept.

The DS-R-J features the items relevant to physical body, while the POPA was found to have multiple subscales that involve psychological purity and physical purity. The subscale categorization of the POPA is reasonable because the correlation between the “Bodily Purity” subscale (i.e., physical purity) of the POPA and the DS-R-J was higher than that between the “Mental Purity” subscale (i.e., psychological purity) and the DS-R-J. The Japanese people's belief in Buddhism can explain why the correlation between the correlation between the “Respect for Religion” of the POPA and the “Dead Body” subscale of the DS-R-J was relatively high. In Buddhism, pollution is believed to be removed by properly mourning for the deceased. Thus, individuals who strongly feel disgust toward the ideas of death and pollution should be more likely depend on Buddhist mourning rituals. The correlation between the POPA and the Animism Scales suggests that the Japanese people's orientation to purity is associated with awe toward the invisible. This orientation involves not just the cleanliness of water and hygiene in a physical sense but also the feeling and respectful attitudes toward something spiritual. This orientation may arise from the Japanese people's respect for the figure like God. However, unlike the Western cultures, the feeling of divinity is not always connected to God because monotheism is not familiar to the Japanese. For the Japanese, the feeling of divinity would be associated with polytheistic and animistic emotions like the naive respect for nature. Because of those attitudes, the “Mental Purity” subscale of the POPA had exclusively a strong correlation with the “Apotheosis of natural products” subscale of the animism scale. Also, the idea that inanimate objects can possess their spirits involved in the “Mental Purity” subscale of the POPA can be interpreted as what the “Anthropomorphization of possessions” subscale of the Animism scale represents. The belief that there are sprits and gods in the nature included in the “Respect for Religion” subscale of the POPA can cause its correlation with the “Apotheosis of natural products” subscale of the animism scale. Further, the Japanese people often bring the objects they possessed for a long time and felt fond of two Buddhist temples and Shinto shrines, where those things undergo some religious rituals and will be properly discarded. That kind of the Japanese people's behavior can explain the relatively strong correlation between the “Respect for Religion” of the POPA and the “Parts of possessors” subscale of the Animism scale.

We examined whether nature worship was associated with familiarity with supernatural phenomena in Study 2. Considering that one's tendency toward POPA is considered to be related to one's pathogen avoidance from an evolutionary perspective, the items in the Pathogen Avoidance subscale were included in POPA. Although this subscale was found to be different from the other subscales in terms of the correlational patterns, it can be part of the moral foundation toward the pure, because uncleanness is often considered immoral (Zhong and Liljenquist, 2006).

## Study 2

Study 2 is an effort (1) to explore the relationship between one's purity orientation and familiarity with supernatural phenomena, (2) to investigate the correlation of the purity items in the MFQ (Graham et al., 2009), and (3) to test the test–retest reliability of the POPA. No particular religion is prevalent in Japan, and most of the Japanese consider religion as something that is associated with superstitious beliefs. As a result, the Japanese population in general is not likely to perceive themselves believing in specific religious beliefs. However, like many people across the world, several Japanese too experience purity and awe by being in proximity with nature, e.g., watching big old trees or wandering and trailing in the woods and mountains. This feeling of awe is not scientific for the Japanese and is considered a wonder, but the feeling of awe has an aspect of reality for the Japanese. Thus, we attempted to find additional validity, investigating the relationship between POPA and Attitudes toward Paranormal Phenomena Scale (APPle), and the relationship between the Mental Purity subscale of POPA and the Purity subscale of the MFQ. Furthermore, the test–retest reliability of the POPA was tested.

## Methods

### Participants

The participants included 199 Japanese undergraduate students enrolled in introductory psychology classes at a major Japanese private university, of whom 167 were males. They were recruited in the classes that included part of students who participated in Study 1. Study 2 was conducted after 2–3 weeks after Study 1. They agreed to participate in this study for extra credit or credit toward a course research requirement. There was no financial incentive provided to the participants. The survey was delivered online with Google Forms. Ethical review and approval by the ethics committee of Toyo University were obtained before conducting this study (P18033). The mean age of the participants was 19.70 (*SD* = 0.97). The procedure was the same as in Study 1.

### Measures

#### Attitudes Toward Paranormal Phenomena Scale Short Version

According to Koshiro et al. (2008), paranormal phenomena are defined as sensations whose existence and effects have not been demonstrated but are believed among people. Koshiro and collaborators developed a scale to measure the Japanese people's attitudes toward such phenomena. The original version has 55 items and six subscales, but later, the authors made it shorter (Sakata et al., 2012). This short version has 30 items with six subscales. Its reliability and validity were found to be sufficient. It has been widely used in previous research conducted in Japan (e.g., Kawakami, 2020). Some sample items include “Psychic power is interesting,” “I am fearful of UFOs,” and “I have seen a ghost.”

#### Moral Foundations Questionnaire

Based on the MFT, Haidt and his colleagues developed the MFQ. It is widely used to measure one's concern for each of the five moral foundations (Graham et al., 2009). We used the 30-item (longer) version of the Japanese MFQ that was back-translated and approved by the authors of the original MFQ (available at www.moralfoundations.org and in Kanai, 2013). The Japanese version of the MFQ was reported to have five factors as the MFT and the original MFQ predicted (Takamatsu and Takai, 2017) and has been used in other research on morality among the Japanese people (e.g., Murayama and Miura, 2019).

## Results and Discussion

The test–retest reliability scores of the four subscales of the POPA were *r* = 0.745, 0.734, 0.699, and 0.744. We then calculated the means of the responses to the items belonging to each subscale for the APPle. Regarding the MFQ, the responses to the items for each moral foundation were summed up. The correlations between the subscales are presented in Table 3.

**Table 3:** Purity orientation–pollution avoidance (POPA) correlations with convergent and discriminant measures (*N* = 199).

**Scale**	**Subscale (*M*, *SD*)**	**Respect for Religion**	**Bodily Purity**	**Pathogen Avoidance**	**Belief in spirituality**	**Preference for fortune telling and magic**	**Skepticism**	**Enjoyment as entertainment**	**Fear**	**Supernatural experience**	**Scale**	**Care**	**Fairness**	**Ingroup**	**Authority**	**Purity**
POPA	Mental Purity (4.08, 1.16)	0.537^**^	0.433^**^	0.230^**^	0.590^**^	0.399^**^	−0.248^**^	0.0601	0.402^**^	0.208^**^	POPA	0.308^**^	0.249^**^	0.374^**^	0.289^**^	0.394^**^
	Respect for Religion (5.37, 1.20)		0.413^**^	0.135	0.510^**^	0.303^**^	−0.291^**^	−0.046	0.219^**^	0.036		0.430^**^	0.388^**^	0.299^**^	0.378^**^	0.455^**^
	Bodily Purity (4.15, 1.09)			0.164^*^	0.182^*^	0.194^**^	−0.0663	0.151^*^	0.192^**^	0.113		0.427^**^	0.307^**^	0.353^**^	0.403^**^	0.497^**^
	Pathogen Avoidance (3.60, 1.23)				0.037	0.126	0.102	0.03	0.0588	0.063		0.083	0.095	−0.024	0.055	0.106
APPle	Belief in spirituality (2.96, 1.07)					0.532^**^	−0.358^**^	0.069	0.474^**^	0.231^**^	APPle	0.123	0.193^**^	0.207^**^	0.225^**^	0.347^**^
	Preference for fortune telling and magic (2.66, 0.95)					−0.305^**^	0.185^**^	0.419^**^	0.323^**^			0.113	0.153^*^	0.238^**^	0.158^*^	0.237^**^
	Skepticism (3.38, 0.94)						0.172^*^	−0.183^**^	−0.233^**^			−0.0586	0.055	−0.143^*^	0.0002	−0.104
	Enjoyment as entertainment (3.18, 0.97)							0.094	0.155^*^			0.169^*^	0.180^*^	0.171^*^	0.190^**^	0.139
	Fear (2.13, 0.88)								0.344^**^			0.026	−0.022	0.200^**^	0.083	0.171^*^
	Supernatural experience (1.42, 0.64)											−0.002	−0.051	0.116	0.125	0.098
MFQ	Care (4.07, 0.79)										MFQ		0.634^**^	0.534^**^	0.374^**^	0.566^**^
	Fairness (3.88, 0.69)													0.386^**^	0.412^**^	0.492^**^
	Ingroup (3.34, 0.76)														0.506^**^	0.498^**^
	Authority (3.36, 0.73)															0.515^**^
	Purity (3.56, 0.72)										

* *p < 0.05*;

** *p < 0.01*.

Among the subscales of the APPle, the “Belief in spirituality” refers to one's tendency to actively believe in supernatural phenomena, and the “Preference for fortune telling and magic” is one's positive attitudes toward the information about fortune telling and the like in mass media and pop culture. These two subscales tap into the core idea of attitudes toward paranormal phenomena. We found a positive correlation between “belief in spirituality” of the APPle and “Mental Purity” of the POPA, *r* = 0.590, which suggests that the psychological aspects of the purity orientation are associated with the familiarity one feels with invisible existence. This correlation was consistent with our expectation and demonstrated the construct validity of the POPA. Similarly, it was reasonable that a moderate positive correlation between the “Preference for fortune telling and magic” of the APPle and the “Mental Purity” of the POPA, *r* = 0.399, was found, given that fortune telling is a practice that embodies customs and traditional religious culture in Japan (e.g., a fortune slip in a Shinto shrine). Compared with the “Mental Purity” subscale, the “Pathogen Avoidance” was not significantly correlated to the two subscales of the APPle mentioned above, probably because one's orientation toward a dirty body, sexual issues, and pathogen-free cleanliness is different from a preference for supernatural phenomena. Therefore, the different correlations between each of the POPA subscales and the other scales show the validity of the POPA as an integrated concept with multiple subscales. Conversely, “Mental Purity” also reflects one's tendency to avoid pollution. Pollution itself is often something one subjectively imagines and is one's feeling that accuses something demonic for mysterious phenomena and natural disasters. People often perceive a supernaturally bad sign from a negative event and fear that something bad would happen, which is part of the purity orientation. That notion of purity orientation can make it reasonable that the positive correlation between the “Fear” subscale of the APPle and the “Mental Purity” of the POPA was observed, *r* = 0.402, to validate the POPA because purity orientation involves not only the enjoyment of supernatural phenomena but also the fear of them. As for the relationship with the MFQ, the subscales of the POPA are positively correlated to almost all the moral foundations, but their correlations with the purity foundation were the strongest. Because of the correlations, a series of *post hoc* multiple regression analyses were performed with the scores for the five moral foundations in the MFQ as the predictors and the scores for the four subscales of the POPA as the outcome variables. The results show that, except for “Pathogen Avoidance,” which had the weakest correlations with the other scales of the POPA, the scores of all the subscales of the POPA were most strongly predicted by the Purity score of the MFQ (Table 4). The findings from the multiple regression analyses demonstrated the relationships between the POPA subscales and the MFQ subscales. The items about harming objects and other people in the “Respect for Religion” and “Bodily Purity” reflect the Japanese strong idea that it is wrong to damage objects and other people, which may be shown in the significant associations between those subscales of the POPA and the Harm subscale of the MFQ.

**Table 4:** Summary of multiple regression analyses for variables predicting purity orientation–pollution avoidance (POPA) scores.

**Criterion variable**	**Predictor**	**β**	***t***	***R*^**2**^**
Mental Purity	Moral foundations questionnaire (MFQ) harm score	0.06	0.59	0.19^**^
	MFQ fairness score	0.01	0.14	
	MFQ ingroup score	0.20	2.28^*^	
	MFQ authority score	0.03	0.40	
	MFQ purity score	0.23	2.63^**^	
Respect for Religion	MFQ harm score	0.22	2.35^*^	0.28^**^
	MFQ fairness score	0.09	1.03	
	MFQ ingroup score	−0.07	−0.86	
	MFQ authority score	0.18	2.29^*^	
	MFQ purity score	0.24	2.83^**^	
Bodily Purity	MFQ harm score	0.22	2.46^*^	0.29^**^
	MFQ fairness score	−0.06	−0.68	
	MFQ ingroup score	0.02	0.20	
	MFQ authority score	0.18	2.32^*^	
	MFQ purity score	0.29	3.46^**^	
Pathogen Avoidance	MFQ harm score	0.08	0.74	0.03
	MFQ fairness score	0.05	0.52	
	MFQ ingroup score	−0.15	−1.63	
	MFQ authority score	0.02	0.27	
	MFQ purity score	0.09	0.96	

* *p < 0.05*;

** *p < 0.01*.

Although purity/pollution involves the purity that religious offerings are believed to have, it is also represented in everyday meals and secular food. The idea that purity plays a crucial role in something ingested from a mouth is observed not only in Japan but also in the West. For example, Jewish and Muslim people do not eat pork because pork is believed to be polluted. Vigilance against food can be, in an evolutionary term, connected to one's psychological tendency of avoiding pathogen infection in the area that he/she resides in. The Purity subscale of the MFQ was not associated with the “Pathogen Avoidance” of the POPA in this study, but the relationship between those two subscales should be observed. The inconsistency may come from the MFQ and represent cultural differences. Cross-cultural comparisons should be awaited in the future to investigate how pathogen avoidance is pronounced in Japan.

The results showed that all subscales except for the “Mental Purity” and “Pathogen Avoidance” of the POPA were moderately correlated to the Purity subscale of the MFQ. This does not suggest that “Mental Purity” and “Pathogen Avoidance” are separate scales, but it means that the Japanese version of the scale is equivalent to the Purity subscale of the MFQ. Since the Purity items in the MFQ are not suitable for the Japanese people's attitudes and emotions involving religiosity and attitudes toward divine existence, the low correlation of the “Mental Purity” and “Pathogen Avoidance” of the POPA and the Purity subscale of the MFQ may have been observed. Douglas (2003) points out that the idea of being cautious about infectious diseases and contagion is observed in the Western and Mediterranean areas with the framework of Judaism and Christianity. Although the concept of pollution can be universally associated with disease infection, the concept is not central to the POPA. It could be possible that the “Mental Purity” and “Pathogen Avoidance” are separate ideas, but both ideas are theoretically relevant to purity. Thus, the “Pathogen Avoidance” subscale should be further scrutinized through future cross-cultural research, taking the validity of the MFQ into consideration. In summary, the validity and reliability of the POPA were demonstrated.

## General Discussion

This work developed the POPA scale and tested its validity and reliability. We included items related to the six factors related to the Japanese concept of purity (i.e., religiosity, death, nature worship, body, sexual issues, and disgust of cleanliness and uncleanness), referring to the characteristics of the Japanese culture. Our factor analysis revealed four components, all of which concerned pollution. The “Pathogen Avoidance” factor includes the items regarding not only sexual issues, such as incest, that are avoided evolutionally but also misdeeds of one's body and sexual relationships and what is considered as a “taboo” in society. The “Mental Purity” factor involves infection avoidance, which is an important aspect of POPA for evolutionary purposes. The “Respect for Religion” factor consists of items about (avoidance of) immoral behavior in the Japanese religious institutions of temples and shrines. These items can be translated and used in the West when the expressions in the items are appropriately modified. For instance, the religious institutions mentioned in the original items would be changed into the church, and a “bad karma” into inviting God's wrath or deviating from God's will.

We specifically focused on the “Mental Purity” factor. The pursuit of mental cleanliness is of late not limited to people in the East because of the latest trend in yoga and oriental meditation. This oriental meditation is widely believed to have an essential effect to purify one's spirit, which allows people to reform bad old customs and accordingly lead a desirable life. In addition to the “Mental Purity” factor, “Respect for Religion” and “Bodily Purity,” involve something people cannot directly perceive or influence their behavior. Meanwhile, the “Pathogen Avoidance” factor is associated with more concrete and familiar concepts (e.g., germs and unsanitary). Regarding the sense of mentality, the “Pathogen Avoidance” factor may be qualitatively different from the other factors, which can explain the different patterns in the correlation with the other factors. Additionally, we found that the “Pathogen Avoidance” subscale of the POPA was not significantly correlated with any foundation of the MFQ. Purity foundations should theoretically include the idea of avoiding pathogens in terms of morality about cleanliness. Considering our findings, it is possible that the items of the MFQ fail to measure the aspect of pathogen avoidance, which suggests that there is still room for improvement. Men participated more than women in Studies 1 and 2. Women were found to score higher in the “Pathogen Avoidance” subscale of the POPA than men, which may indicate the possibility that there are gender differences in how much the subscale has effects on other variables. This finding may be consistent with some previous research that showed sex differences in disgust sensitivity and the fear of contamination, with women scoring higher than men (Haidt et al., 1993; Arrindell et al., 1999; Olatunji et al., 2005). More equal sample collection of men and women to investigate whether the present research is replicable is needed for future research.

The concept of one's purity orientation can be applied to research on various contexts, such as discrimination against outgroup members and (former) criminals, and the attitudes toward immigrants, radioactive contamination, and political corruption. Future research can investigate the possibility that attitudes toward outgroups depend not only on Pathogen Avoidance in a physical sense but also on purity orientation in a psychological sense. What regulates the relationship between purity orientation and these factors will be a prospective direction to scrutinize. In addition, cross-cultural comparisons regarding purity orientation would be of interest for future research. A cross-cultural approach can evaluate possible relationships between purity orientation, and self-construal and relational mobility and can further identify social and ecological factors to intensify one's purity orientation. Overall, POPA is a promising tool to contribute to future research, not only in psychology but also in other academic areas, such as folklore and sociology. This work contributes to moral psychology to better understand the complicated mechanisms of purity, its function, and its relationships with other concepts.

## Data Availability Statement

The raw data supporting the conclusions of this article are made available at https://osf.io/keuwd/.

## Ethics Statement

The studies involving human participants were reviewed and approved by Ethics Committee, Department of Sociology, Toyo University. The patients/participants provided their written informed consent to participate in this study.

## Author Contributions

All authors listed have made a substantial, direct and intellectual contribution to the work, and approved it for publication.

## Conflict of Interest

The authors declare that the research was conducted in the absence of any commercial or financial relationships that could be construed as a potential conflict of interest.
